# Monocyte‐related cytokines/chemokines in cerebral ischemic stroke

**DOI:** 10.1111/cns.14368

**Published:** 2023-07-14

**Authors:** Meiling Bai, Ruize Sun, Bin Cao, Juan Feng, Jue Wang

**Affiliations:** ^1^ Department of Neurology Shengjing Hospital of China Medical University Shenyang China

**Keywords:** chemokine, cytokine, inflammation, ischemic stroke, monocyte

## Abstract

**Aims:**

Ischemic stroke is one of the leading causes of death worldwide and the most common cause of disability in Western countries. Multiple mechanisms contribute to the development and progression of ischemic stroke, and inflammation is one of the most important mechanisms.

**Discussion:**

Ischemia induces the release of adenosine triphosphate/reactive oxygen species, which activates immune cells to produce many proinflammatory cytokines that activate downstream inflammatory cascades to induce fatal immune responses. Research has confirmed that peripheral blood immune cells play a vital role in the immunological cascade after ischemic stroke. The role of monocytes has received much attention among numerous peripheral blood immune cells. Monocytes induce their effects by secreting cytokines or chemokines, including CCL2/CCR2, CCR4, CCR5, CD36, CX3CL1/CX3CR1, CXCL12(SDF‐1), LFA‐1/ICAM‐1, Ly6C, MMP‐2/9, NR4A1, P2X4R, P‐selectin, CD40L, TLR2/4, and VCAM‐1/VLA‐4. Those factors play important roles in the process of monocyte recruitment, migration, and differentiation.

**Conclusion:**

This review focuses on the function and mechanism of the cytokines secreted by monocytes in the process of ischemic stroke and provides novel targets for treating cerebral ischemic stroke.

## Graphical abstract

In this review, we discuss cytokines, chemokines and their receptors, related to the recruitment and migration of monocytes in cerebral ischemic stroke. This article helps to better understand the role of these molecules in ischemic stroke and develop new treatment strategies.

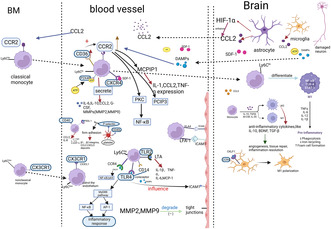

## 1 INTRODUCTION

Ischemic stroke is caused by a reduction in cerebral blood flow and is a leading cause of death worldwide.^1^ It is the most common cause of disability in Western countries^2^ with a significant impact on older adults, especially those with comorbidities (hypertension, diabetes mellitus, and other chronic diseases), and can leave patients with different levels of disability (e.g., hemiplegic paralysis), leading to difficulties in daily activities.

Acute brain ischemia, which leads to insufficient oxygen/glucose/lipid supply to the brain, can cause loss of cell integrity and trigger apoptosis/necrosis,^1^ resulting in neuronal death.^3^ Multiple mechanisms contribute to ischemic brain injury, including excitotoxicity, oxidative stress, and inflammation.^2^ In the central nervous system (CNS), ischemic neuronal injury results in a significant release of glutamate, which causes excessive activation of *N*‐methyl‐D‐aspartate receptors and a massive influx of calcium ions, leading to cell death due to excitotoxicity.^2^ Dying cells and cell debris can cause a series of inflammatory responses mediated by cytokines (e.g., interleukins [ILs] and tumor necrosis factor‐alpha [TNF‐α]), chemokines (e.g., C‐C motif chemokine ligand [CCL] 2 [also known as monocytic chemotactic protein‐1 (MCP‐1)]), and the activation of immune cells (both resident and peripheral immune cells), leading to neuronal death. Resident immune cells, such as astrocytes and microglia, secrete cytokines and chemokines that create a proinflammatory environment. Astrocytes and damaged neurons can also produce reactive oxygen species (ROS), which deplete glutathione, an essential antioxidant that prevents ROS‐mediated DNA damage,^2^, ^4^ which attracts peripheral immune cells to the ischemic area to initiate downstream inflammatory cascades. After the accumulation of activated immune cells, microglia are activated by an increase in extracellular adenosine triphosphate (ATP) from the depolarization of neurons and glia and the release through damaged plasma membranes of dying cells. Activated microglia secrete proinflammatory mediators, such as cytokines (e.g., TNF‐α), and develop phagocytic and major histocompatibility complex class II‐restricted antigen‐presenting characteristics. Microglial activation enhances the production of growth factors, such as brain‐derived neurotrophic factor (BDNF), and removes dead cells and cell debris.^2^ Astrocytes and microglia can draw peripheral immune cells into the ischemic area. In circulation, the proinflammatory cytokines and chemokines produced by immune cells contribute to the destruction of the blood–brain–barrier (BBB). Matrix metalloproteinases (MMPs) cause BBB and extracellular matrix (ECM) degradation, resulting in brain edema and cell/cytokine leakage that can damage infarcted tissue.^5^ Astrocyte‐secreted hypoxia‐inducible factor‐1‐alpha (HIF‐1α) promotes the secretion of CCL2, which attracts monocytes to the brain via the bloodstream.^6^, ^7^

Peripheral immune cells, especially monocytes, are involved in the inflammatory response. Monocyte recruitment to the brain causes an inflammatory response that promotes brain damage. Inflammation and ischemic brain injury are strongly connected, and inflammation is associated with stroke severity and outcome.^8^ Stroke causes the excessive release of ATP/ROS, which are sensed by immune cells through the purinergic receptor P2X4 (P2X4R).^9^ Activated immune cells produce cytokines and chemokines that participate in the next step of the inflammatory response. Monocytes are heterogeneous white blood cells that circulate in the bloodstream and differentiate into macrophages or dendritic cells (DCs) depending on the local tissue environment.^10^ Traditionally, monocytes are divided into three subsets: classical, intermediate, and non‐classical. Classical monocytes have a proinflammatory role in the inflammatory response, whereas non‐classical monocytes patrol the endothelium and have an anti‐inflammatory role.^11^ In ischemic stroke, cell surface pattern‐recognition receptors initiate an inflammatory response after sensing damage‐associated molecular patterns (DAMPs) by activating immune cells, including monocytes.^8^ C‐C motif chemokine receptor (CCR) 2 is expressed on classical monocytes. CCL2 binds to CCR2 to mediate monocyte migration into the brain. The main subset of monocytes recruited to the brain is the classical subset, which plays a proinflammatory role in the acute phase, producing cytokines, such as IL‐6 and TNF‐α.^12^, ^13^ Toll‐like receptors (TLRs) are expressed on monocytes and can enhance the production of IL‐1β/6, CCL2, and TNF‐α.^14^ CCL2 production forms a positive feedback loop that promotes monocyte recruitment to the ischemic hemisphere, enhancing the inflammatory response.^15^ CCL2 also regulates P2X4R expression, which is stimulated by excessive ATP production.^16^ Intercellular adhesion molecules (ICAMs) and vascular cell adhesion molecules (VCAM) 1, both of which are expressed in endothelial cells, mediate monocyte rolling and adhesion to regulate the patrolling behavior of non‐classical monocytes. VCAM‐1 binding to its receptor, very late activation antigen (VLA) 4, which is expressed on monocytes, is associated with monocyte migration.^17^ CCL2 can mediate the conformation of VLA‐4 to display a high affinity for VCAM‐1, which promotes monocyte migration.^18^ According to the “dead cell clearance hypothesis,” once recruited to the ischemic area, classical monocytes may acquire the M2 phenotype and play an anti‐inflammatory role.^19^ Thus, monocytes may be beneficial in ischemic stroke. Circulating monocyte depletion in a mouse model was associated with a decrease in the expression of anti‐inflammatory factor genes and the number of monocyte‐derived macrophages (MDMs), which impairs long‐term neurological recovery.^20^ M2 macrophages produce anti‐inflammatory cytokines and are associated with angiogenesis, which may facilitate recovery from disability after ischemic stroke.

Several factors, from monocyte migration to cytokine activation and secretion, are involved in the pathophysiology of ischemic stroke. CCL2 binds to CCR2 to mediate monocyte migration. Stroke severity is associated with the level of CCR2 expression and the number of infiltrated monocytes. C‐X3‐C motif chemokine ligand (CX3CL) 1 (also known as fractalkine) and C‐X3‐C motif chemokine receptor (CX3CR) 1 (also known as G protein‐coupled receptor 13) are required for the patrolling behavior of non‐classical monocytes, among other functions.^21^ C‐X‐C motif chemokine ligand (CXCL) 12 (also known as a stromal cell‐derived factor [SDF] 1) regulates the late accumulation of monocytes in the ischemic hemisphere. Nuclear receptor subfamily 4 group A member 1 (NR4A1) is responsible for the generation of lymphocyte antigen 6C (Ly6C)^−^ monocytes and regulates neuroinflammation via the nuclear factor‐kappa B (NF‐κB) pathway and monocyte–macrophage differentiation. MMPs are involved in the secretion of proinflammatory cytokines. P‐selectin and CD40L play essential roles in monocyte–platelet interactions. TLRs, especially TLR4, which is a coreceptor for CD14 on monocytes, are involved in the recognition of stimulatory signals and trigger the activation of inflammatory pathways.^22^, ^23^

In this review, we aim to highlight the role of monocytes in ischemic stroke and provide new targets for the treatment of cerebral ischemic stroke. Here, we discuss the role and function of monocyte‐secreted cytokines/chemokines in ischemic stroke. We also describe the classification of monocytes, their role in ischemic stroke, and the cytokines/chemokines secreted by monocytes and their function.

### 1.1 Monocyte classification

Human monocytes can be divided into three subsets based on cell surface antigen (CD14, a component of the lipopolysaccharide [LPS] receptor complex, and CD16, the FCγRIII immunoglobulin receptor) expression: classical (CD14^++^CD16^−^), intermediate (CD14^+^CD16^++^), and non‐classical (CD14^+^CD16^++^).^11^ Classical monocytes express CCR2 and low levels of CX3CR1, whereas non‐classical monocytes do not express CCR2 but express high levels of CX3CR1. Murine monocytes can be divided into two subsets based on Ly6C expression: CCR2^+^CX3CR1^lo^, which express high levels of Ly6C and are considered classical/proinflammatory; and CCR2^−^CX3CR1^hi^, which express low levels of Ly6C and are considered non‐classical with patrolling behavior along the endothelium.^1^

Monocytes can also be classified according to TIE2 and SLAN expression. TIE2^+^ monocytes have proangiogenic properties that promote tumor angiogenesis, while “regulation of cytokine production” is the key gene ontology term that distinguishes SLAN^+^ from SLAN^−^ monocytes. Interaction analysis demonstrated that a cluster of Ubiquitin C‐related genes, which regulate diverse cellular processes, is highly expressed in SLAN^+^ monocytes.^24^ CD16^+^ monocytes can be reclassified according to their relative expression of TIE2 and SLAN into TIE2^+^SLAN^−^, TIE2^−^SLAN^+^, and TIE2^−^SLAN^−^ subsets.^25^ Damasceno et al. (2019) identified five distinct subsets of monocytes based on L‐selectin (CD62L) and SLAN expression: CD62L^+^ classical, CD62L^−^ classical, intermediate, SLAN^+^ non‐classical, and SLAN^−^ non‐classical.^26^

**FIGURE 1:**
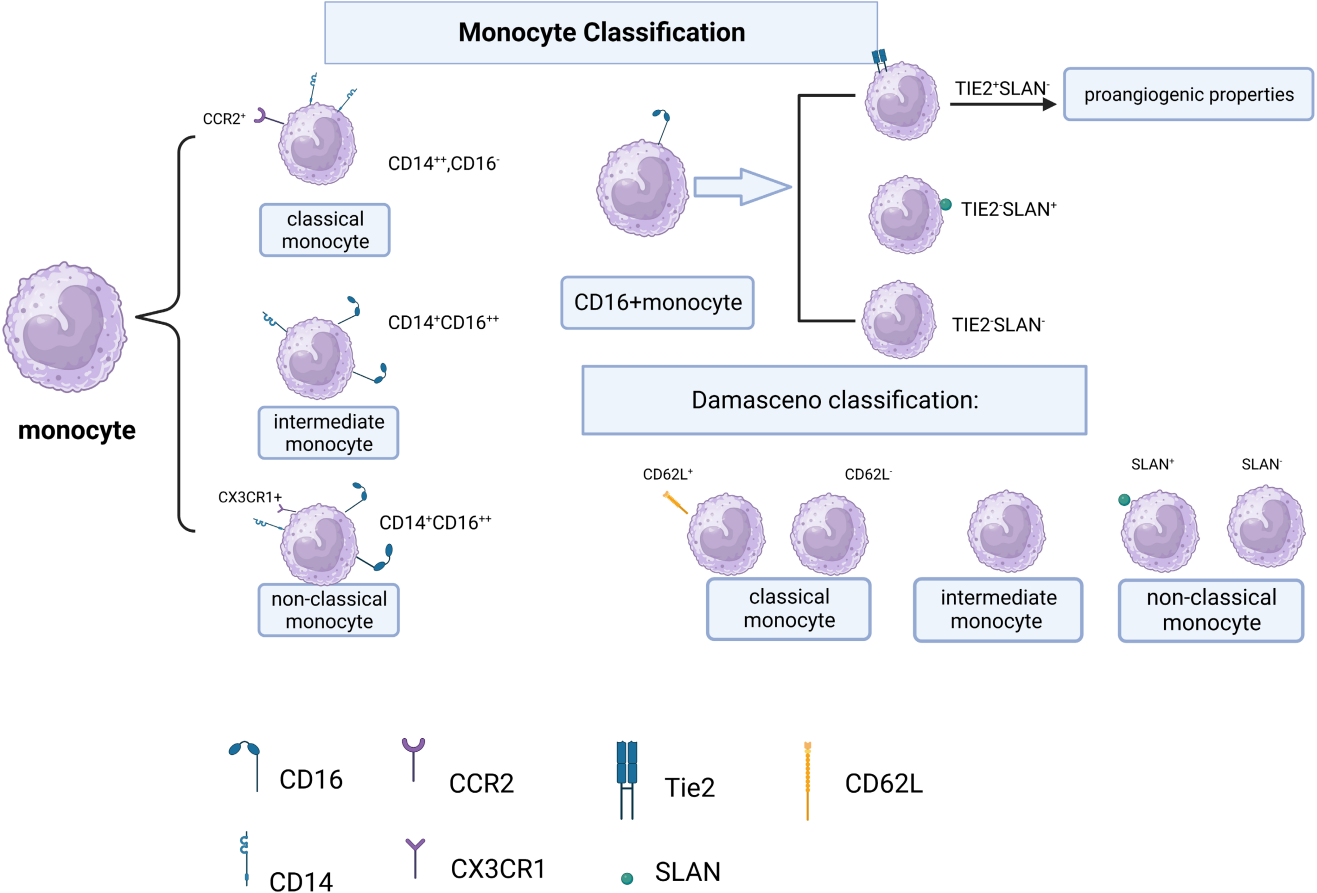
Classification of monocytes. Tie2: TEK tyrosine kinase/CD202b.

Classical monocytes are scavenger cells that phagocytose endogenous and exogenous pathogens (dead cells, cell debris, and bacteria). They are proinflammatory and produce the highest levels of CCL2, granulocyte colony‐stimulating factor (CSF), and IL‐6/10 among the three subsets.^25^, ^27^ They also produce high levels of ROS and the highest levels of CCL2/3 and IL‐8/10 in response to LPS stimulation.^24^ Intermediate monocytes are in transition from classical to non‐classical monocytes. They produce steady‐state IL‐1β and TNF‐α, are anti‐inflammatory, have phagocytic activity, but not as high as classical monocytes, and are the main producers of ROS.^11^, ^25^ They also produce the highest levels of IL‐1β/6 and TNF‐α in response to LPS stimulation.^24^ Non‐classical monocytes provide immune surveillance by patrolling the endothelium. They are responsible for CD4^+^ T‐cell proliferation and can stimulate IL‐4 production by CD4^+^ T cells.^25^ They also produce IL‐1β and TNF‐α but do not generate ROS^24^, ^27^ The classification of monocytes is in Figure 1.

## 2 ROLE OF MONOCYTES IN ISCHEMIC STROKE

### 2.1 Monocyte recruitment during ischemic stroke

Monocytes are recruited to the ischemic hemisphere immediately after an ischemic stroke. Inflammation is initiated by pattern‐recognition receptors that recognize DAMPs (modified/oxygenized lipids, cytoplasmic proteins, DNA, and RNA) released by resident immune cells, such as microglia, which sense ATP released by damaged cells and migrate to the damaged area.^1^, ^8^ P2X4R is an ATP receptor and a downstream target of CCL2, which is involved in monocyte recruitment. Microglia secrete proinflammatory cytokines, such as IL‐1β and TNF‐α, to create an inflammatory environment that attracts immune cells.^1^

Monocyte recruitment is mediated by the interaction of monocytes with the endothelium via adhesion molecules (integrins, selectins, and immunoglobulins).^28^, ^29^ Activated classical monocytes enter the site of inflammation by adhering to the endothelial surface through interactions with integrins and other adhesion molecules in the bloodstream and infiltrating the vascular walls. Adhesion molecules expressed by classical monocytes that are involved in this process include CD62L, lymphocyte function‐associated antigen (LFA)‐1, macrophage receptor 1, platelet endothelial cell adhesion molecule 1, P‐selectin glycoprotein ligand 1 (PSGL‐1), and VLA‐4.^29^ VLA‐4/VCAM‐1 have also been shown to be involved in Ly6C^lo^ monocyte recruitment to the brain in an infection model^1^, ^28^ However, whether Ly6C^lo^ monocyte recruitment is involved in ischemic stroke remains unclear. Activated classical monocytes can alter the conformation of VLA‐4 via the CCL2/CCR2 axis, thereby leading to higher VCAM‐1 binding affinity and monocyte migration to the infarcted tissue.^18^

CCL2/CCR2 are involved in monocyte recruitment to the ischemic brain. CCR2‐deficient mice have reduced classical monocyte recruitment.^1^, ^30^ CCL2 mediates monocyte migration from the bone marrow into the bloodstream by binding to CCR2, whose expression is associated with stroke severity. Many cytokines can regulate CCL2 expression to mediate monocyte migration, including CD36, HIF‐1α, MMPs, and P‐selectin.^6^, ^7^, ^31^, ^32^, ^33^ Monocyte migration can also be induced by granulocyte–macrophage CSF. SDF‐1 has been shown to play a role in the homing of microglia/monocytes/macrophages/stem cells, which mainly function in the later phase of monocyte recruitment, to areas of ischemic injury.^34^ Classical monocytes are also recruited to the site of inflammation by the release of CD73 (ecto‐5′‐nucleotidase), CXCL1, interferon (IFN)‐gamma (IFN‐γ), and mucosal addressing cell adhesion molecule 1.

### 2.2 BBB disruptions

Monocytes can disrupt the BBB. Classical monocytes recruited to the brain act as proinflammatory mediators. M1 monocytes secrete ROS and cytokines/chemokines through inflammasome activation, which degrade tight junctions (TJs) between endothelial cells and the BBB.^19^ In experimental stroke, blocking CCR2 to inhibit monocyte recruitment protects against brain edema but impairs long‐term recovery.^19^, ^35^ Monocytes exacerbate secondary inflammatory BBB damage by upregulating the triggering receptor expressed on myeloid cells 1, which activates other innate immune receptors and promotes inflammatory cytokine/chemokine production, including IL‐8, MCP‐1/3, and macrophage inflammatory protein‐1 alpha (MIP‐1α).^19^, ^36^ Monocytes also upregulate adipocyte fatty acid‐binding protein expression to potentiate MMP‐9‐mediated degradation of TJs by enhancing c‐Jun *N*‐terminal kinase (JNK)/c‐Jun signaling.^19^, ^37^ The function and phenotype of monocytes vary during ischemic stroke. Monocytes switch from proinflammatory M1 dominant on Day 3 to anti‐inflammatory M2 dominant on Day 7 after stroke, indicating the functional transformation of monocytes from enhancing the immune response to the resolution of inflammation.^19^

The role of monocytes in the BBB in ischemic stroke remains controversial. Some studies have suggested that monocytes disrupt the BBB as increased numbers of circulating monocytes are associated with the hyperintense acute reperfusion marker, a larger acute ischemic stroke (AIS) volume, worse National Institutes of Health Stroke Scale (NIHSS) scores, and poorer outcomes.^38^ However, other studies support the opposite view, as monocyte counts did not change during AIS and could not predict long‐term mortality.^39^ These opposing views may be due to the classification of monocytes; classical and non‐classical monocytes play different roles in ischemic stroke. Lower non‐classical monocyte counts are associated with poorer outcomes, suggesting a protective role of non‐classical monocytes in ischemic stroke.^19^

### 2.3 Monocyte–macrophage differentiation

The monocytes recruited to the ischemic brain are mostly Ly6C^hi^CCR2^+^ monocytes, which differentiate into M1 macrophages in the ischemic hemisphere and promote inflammation.^12^ Evidence suggests that Ly6C^hi^ monocytes can differentiate into both M1 and M2 macrophages.^1^ Proinflammatory M1 macrophages produce IL‐6 and TNF‐α and promote tissue degradation.^18^ Anti‐inflammatory M2 macrophages produce anti‐inflammatory cytokines, such as IL‐10, and promote angiogenesis/tissue repair.^18^ M2 macrophages can be divided into three subsets: M2a, M2b, and M2c. M2a macrophages are induced by IL‐4/13. IL‐13 promotes the transformation of macrophages from the M1 to the M2a phenotype in mice 3 days after permanent ischemia, enhances the anti‐inflammatory response of M2 macrophages, and reduces the number of apoptotic neurons.^19^, ^40^ Park et al.^41^ reported that half of monocytes differentiate into proinflammatory M1 macrophages, and the other half differentiate into anti‐inflammatory M2 macrophages. Recruited infiltrating Ly6C^hi^ monocytes downregulate Ly6C expression and upregulate macrophage biomarker F4/80 expression.^1^ They progressively acquire the expression of typical markers of alternatively activated macrophages, such as ARG‐1 and YM‐1.^42^ During the subacute phase, when they persist at sites of inflammation, infiltrating Ly6C^hi^ monocytes lose CCR2 and Ly6C expression and start to express transforming growth factor‐beta (TGF‐β) and vascular endothelial growth factor (VEGF) and TGF‐β to promote angiogenesis/neuroprotection.^12^, ^43^ Some researchers have referred to this phenomenon as the “dead cell clearance” hypothesis, which states that exposure to apoptotic cells causes classically activated M1 macrophages to polarize to the M2 phenotype.^12^, ^44^ This suggests that the role of proinflammatory monocytes may vary during the subacute phase of stroke and may be beneficial for tissue repair in the subsequent resolution phase. Conventionally, proinflammatory monocytes differentiate into M1 macrophages and anti‐inflammatory monocytes differentiate into M2 macrophages. However, studies have shown that, even in the absence of anti‐inflammatory monocytes, M2 macrophages can be found in the infarcted brain, indicating that monocyte–macrophage differentiation mainly occurs in classical monocytes.^45^

The following functions have been proposed for MDMs in ischemic stroke: MDMs have a dual effect on the progression of ischemic stroke. M1 macrophages promote inflammation while M2 macrophages suppress inflammation and stimulate tissue repair.^8^ MDMs (not microglia) are the major phagocytes in ischemic stroke. MDMs have the highest phagocytic activity among the different cell types in the post‐ischemic brain. Phagocytosis is required for the conversion of CD45^hi^ MDMs to a CD45^lo^ microglia‐like phenotype.^46^ MDM depletion in the ischemic brain of CCR2 knockout (KO) mice results in a smaller infarct size 3 days after stroke but is associated with greater injuries, higher mortality, and reduced functional recovery 14 and 28 days after stroke.^35^ Evidence suggests that the ratio of M1 to M2 MDMs shifts toward the proinflammatory phenotype on Day 3 and the anti‐inflammatory phenotype thereafter. MDMs are the main source of BDNF and TGF‐β, which contribute to long‐term recovery from stroke.^20^ The role of macrophages in ischemic stroke is changeable, and the timing of intervention may be crucial for the treatment of ischemic stroke.

### 2.4 Inflammatory and anti‐inflammatory roles of monocytes in ischemic stroke

Once recruited to the ischemic hemisphere, classical monocytes secrete proinflammatory cytokines, including IL‐1/6 and TNF‐α, which promote inflammation, affect the infarcted tissue, and aggravate ischemic injury in ischemic stroke. Monocytes trigger downstream responses related to CCL2, such as the activation of MCP‐1‐induced protein‐1 and the expression of IL‐1 and TNF genes, which promote inflammation through the interaction of CCL2 with CCR2.^47^, ^48^ CCL2 activates the downstream target P2X4R, which has a dual effect on the progression of ischemic stroke. On the one hand, the production of proinflammatory cytokines is associated with P2X4R, and, on the other hand, P2X4R^+^ cells produce BDNF, which contributes to the recovery of ischemic stroke.^49^ Classical monocytes secrete MMP‐2/9, which degrades the TJs of the BBB, resulting in cell leakage and ECM degradation, leading to edema and ischemic brain injury.

**FIGURE 2:**
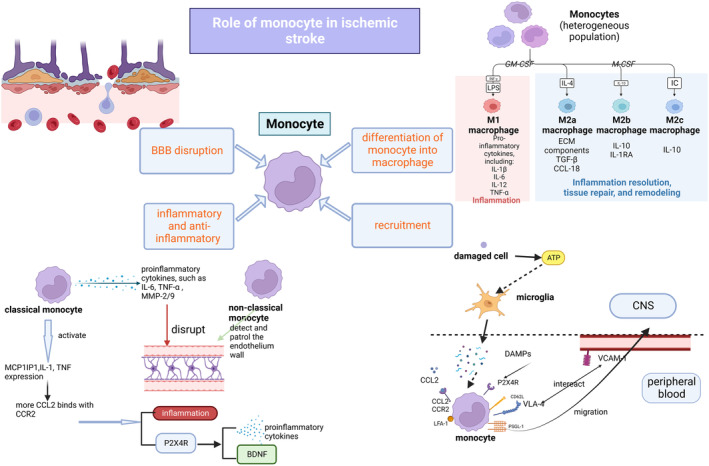
Role of monocyte in ischemic stroke. ATP, adenosine triphosphate; CCL, C‐C motif chemokine ligand; CCR, C‐C motif chemokine receptor; CNS, central nervous system; DAMPs, damage‐associated molecular patterns; G‐CSF, granulocyte colony‐stimulating factor; GM‐CSF, Granulocyte–macrophage colony‐stimulating factor; LFA‐1, lymphocyte function‐associated antigen 1; LPS, lipopolysaccharide; MCP1P1, monocyte chemoattractant protein‐1 induced protein 1; M‐CSF, macrophage colony‐stimulating factor; P2X4R, purinergic receptor P2X4; PSGL‐1, P‐selectin glycoprotein ligand 1; TGF‐β, transforming growth factor‐beta; TNF‐α, tumor necrosis factor‐alpha; VLA‐4, very late antigen 4.

Classical monocytes are the main subset recruited to the ischemic brain. They downregulate CCR2 and differentiate into non‐classical monocytes.^45^ Non‐classical monocytes can detect the integrity of the endothelial wall, through crawling behavior, and produce anti‐inflammatory cytokines, such as IL‐10, for which monocytes are the main producers. IL‐10 suppresses the excessive production of proinflammatory cytokines and may be beneficial for the recovery of ischemic stroke. IL‐10‐deficient mice had larger damaged areas, greater brain atrophy, and poorer long‐term outcomes in a model of transient middle cerebral artery occlusion (MCAO).^50^ Figure 2 shows a schematic of the role of monocytes in ischemic stroke.

## 3 MONOCYTE‐RELATED CYTOKINES/CHEMOKINES IN ISCHEMIC STROKE

**FIGURE 3:**
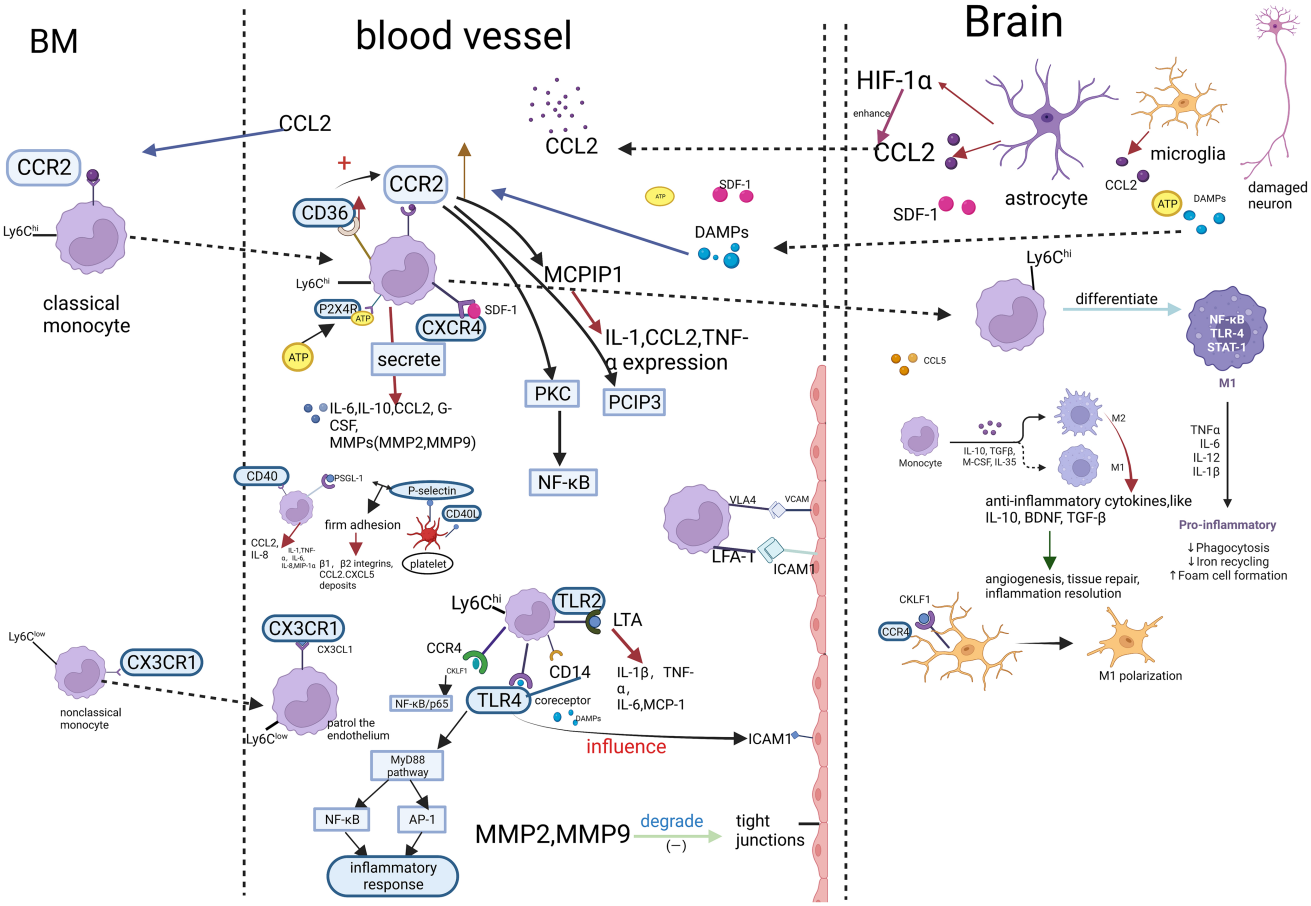
Cytokines/chemokines involved in the recruitment and migration of monocytes in ischemic stroke. ATP, adenosine triphosphate; BDNF, brain‐derived neurotrophic factor; BM, bone marrow; CCL, C‐C motif chemokine ligand; CCR, C‐C motif chemokine receptor; CKLF1, chemokine‐like factor 1; CX3CL, C‐X3‐C motif chemokine ligand; CX3CR, C‐X3‐C motif chemokine receptor; CXCL, C‐X‐C motif chemokine ligand; CXCR, C‐X‐C motif chemokine receptor; DAMP, damage‐associated molecular pattern; G‐CSF, granulocyte colony‐stimulating factor; HIF‐1α, hypoxia‐inducible factor‐1‐alpha; ICAM, intercellular adhesion molecule‐1; IL, interleukin; LFA, lymphocyte function‐associated antigen 1; Ly6C, lymphocyte antigen 6C; MCP‐1, monocyte chemotactic protein‐1; MCPIP1, monocyte chemoattractant protein‐1 induced protein 1; M‐CSF, macrophage colony‐stimulating factor; MMP, matrix metalloproteinase; MyD88, myeloid differentiation factor 88; NF‐κB, nuclear factor‐kappa B; P2X4R, purinergic receptor P2X4; PKC, protein kinase C; PSGL‐1, P‐selectin glycoprotein ligand‐1; SDF, stromal cell‐derived factor; STAT1, signal transducer and activator of transcription 1; TGF‐β, transforming growth factor‐beta; TLR, Toll‐like receptor; TNF‐α, tumor necrosis factor‐alpha; VCAM, vascular cell adhesion molecule; VLA, very late activation antigen.

Under the circumstance of ischemic stroke, monocytes secret several cytokines and chemokines. They could help in the recruitment, migration, and differentiation of monocytes. The monocyte‐related cytokines and chemokines in ischemic stroke include CCL2/CCR2, CCR4, CCR5, CD36, CX3CL1/CX3CR1, CXCL12(SDF‐1), LFA‐1/ICAM‐1, Ly6C, MMP‐2/9, NR4A1, P2X4R, P‐selectin, CD40L, TLR2/4, VCAM‐1/VLA‐4, and their functions are listed in Table 1 below. Figure 3 shows the cytokines/chemokines involved in the recruitment and migration of monocytes in ischemic stroke.

**TABLE 1:** Name, localization, and function of monocyte‐related cytokines/chemokines and their role in ischemic stroke.

Name	Localization	Function in monocyte	Function in ischemic stroke	References
CCL2 (MCP‐1)/CCR2	CCR2 is expressed in monocytes. CCL2 is secreted by many cells, including astrocytes, endothelial cells, neurons, and pericytes	Monocyte migration from the bone marrow to the blood. Mediates monocyte migration to inflamed tissue	Enhances the proinflammatory function of monocytes via P2X4R and promotes long‐term recovery of ischemic stroke. CCL2 promotes leukocyte crossing of the BBB and alters the expression of TJs in BMECs. CCL2/CCR2 binding induces conformational changes in VLA‐4. CCR2^+^ monocytes express high levels of VEGF‐A and can mediate the expression of PDGF beta and PDGF receptor beta mRNA in the ischemic state	[9, 15, 16, 18, 48, 52, 53, 54, 55, 56, 57, 65, 66, 67, 68]
CCR4	Expressed on monocytes and T cells	May play a role in monocyte migration	CCR4 plays an important role in the polarization of macrophages/microglia	[73, 74, 75]
CCR5	Expressed on intermediate monocytes	Mediates the migration of monocytes/MDMs	CCL5/CCR5 expression is increased in the ischemic hemisphere, which mediates the recruitment of Ly6C^+^ monocytes	[41, 74, 78]
CD36 (a highly glycosylated class B scavenger receptor)	Expressed in many cell types, including monocytes, macrophages, and microglia	Interacts with ligands to initiate inflammatory responses. Enhances the phagocytosis ability of microglia and monocytes/macrophages	CD36 cell surface expression is upregulated. CD36 may regulate monocyte recruitment by regulating CCL2/CCR2 expression. CD36 exacerbates brain injury in transient MCAO. CD36 may have a negative effect on the early phase of ischemic stroke, and reparative functions in the resolution phase	[32, 82, 84, 85]
CD40L (a transmembrane protein)	Expressed on activated platelets	Triggers the expression of adhesive molecules, such as E/P‐selectin and ICAM‐1, leading to the formation of platelet–leukocyte aggregates. Interacts with CD40 to stimulate IL‐1/6/8, TNF‐α, and MIP‐1α secretion in thrombosis	CD40L levels increase during the acute/subacute phase. CD40L expression is higher in small‐artery disease stroke than in cardioembolic stroke	[111, 115, 116, 117]
CX3CL1/CX3CR1	CX3CL1 is produced by neurons. CX3CR1 is highly expressed in non‐classical monocytes	Required for the patrolling behavior of non‐classical monocytes. Activates integrins and has intrinsic adhesion properties	CX3CR1 may impact the volume of the infarcted area, the integrity of the BBB, angiogenesis, and neurological recovery	[21, 86, 87, 92, 93, 94]
CXCL12 (SDF‐1)	Produced by activated astrocytes	Regulates monocyte adhesion to BMECs	Recruits monocytes to infarcted tissue during the late stages of stroke	[34, 96]
ICAM‐1/LFA‐1	ICAM‐1 is expressed on endothelial cells; LFA‐1 is expressed on monocytes	Required for the patrolling behavior of Ly6C^−^ monocytes in the endothelium	Soluble ICAM‐1 is elevated, and high serum ICAM‐1 levels are associated with poor outcome	[18, 21, 97]
Ly6C	Highly expressed in classical monocytes	Marker of classical monocytes	Ly6C^hi^ monocytes are involved in macrophage polarization.^98^ Ly6C^hi^ monocytes differentiate into M1 macrophages and secrete inflammatory cytokines. Ly6C^lo^ monocytes have little impact on ischemic stroke	[1, 95, 98]
MMP‐2	Secreted by monocytes	Involved in the secretion of chemokines, such as CCL2. Elevated during monocyte‐to‐macrophage differentiation	Early elevated MMP‐2 levels may be associated with better outcomes. Negative correlation between plasma MMP‐2 levels on admission and NIHSS scores. Patients with mild and improving symptoms have higher MMP‐2 levels. MMP‐2 is associated with BBB dysfunction and leukoaraiosis	[31, 101, 102, 103]
MMP‐9	Secreted by monocytes	Involved in monocyte activation by interacting with soluble CD14, reducing monocyte responsiveness to LPS. Adipocyte fatty acid‐binding protein enhances JNK/c‐Jun activation, promoting MMP‐9 transactivation in peripheral monocytes (macrophages and microglia) and accelerating the breakdown of the BBB	Induces proinflammatory cytokines and chemokines, including CXCL8, IL‐1β, and TNF‐α. MMP‐9 damages BBB integrity by degrading TJs and the ECM to promote cerebral edema. Late elevated MMP‐9 levels are associated with poor outcome, whereas reduced MMP‐9 levels on admission are associated with better NIHSS scores	[5, 31, 101, 103]
NR4A1	Expressed on monocytes	Essential for Ly6C^−^ monocyte survival and the transition from Ly6C^+^ to Ly6C^−^ monocytes. May be involved in monocyte‐to‐macrophage differentiation	Regulates neuroinflammation in cerebral ischemia by interacting with NF‐κB/p65. May be involved in microglial M1 polarization and CCL2/7 secretion	[11, 41, 101, 107, 108]
P2X4R	Highly expressed in immune cells	Activation of P2X4R^+^ cells, especially myeloid cells, releases proinflammatory cytokines, including IL‐1β/6 and TNF‐α, and may promote monocyte migration to the brain	P2X4R activation induces BDNF release, promoting monocyte polarization which secretes proinflammatory cytokines and chemokines and degrades TJs between endothelial cells and BBB	[9, 16, 19, 49]
P‐selectin	Resides in the alpha‐granule membrane of unstimulated platelets	Monocyte–platelet tethering. Binds to PSGL‐1 expressed on monocytes. Promotes monocyte secretion of CCL2, IL‐8, and TNF‐α, after tight adhesion, and deposition of platelet chemokines (CCL5/CXCL4)	Elevated P‐selectin levels during the acute/subacute phase are associated with ischemic stroke severity and outcome	[33, 87, 111, 112, 113, 114]
TLR2/TLR4	TLR2/TLR4 are expressed in innate immune cells, such as monocytes	Recognize cell debris, pathogens, and microbial products to trigger an inflammatory response. TLR2 recognizes LTA and peptidoglycan; TLR4 recognizes LPS. TLR2 induces the production of proinflammatory cytokines (CCL2, IL‐1β/6, and TNF‐α). CD14 is a coreceptor for TLR4 (highly expressed on classical monocytes). CD14/TLR4 sense DAMPs	Increased TLR2 expression is associated with poor outcome, neuronal death, and the accumulation of inflammatory cells, including monocytes. TLR2 requires CD36 to initiate an inflammatory response in ischemia. TLR4 is associated with stroke severity and cytokine/ICAM‐1 levels. TLR4 can activate multiple downstream inflammatory pathways to trigger proinflammatory gene expression	[14, 22, 23, 118, 119, 121, 123, 126, 127, 128]
VLA‐4/VCAM‐1	VLA‐4 is expressed in monocytes. VCAM‐1 is expressed in endothelial cells	Rolling, adhesion, and transmigration of monocytes. Blocking VLA‐4/VCAM‐1 reduces monocyte recruitment to the brain vasculature during infection	VCAM‐1 is elevated from stroke onset to the chronic phase. Soluble VCAM‐1 is associated with short‐term mortality	[17, 130, 131]

Abbreviations: BBB, blood–brain–barrier; BDNF, brain‐derived neurotrophic factor; BMEC, brain microvascular endothelial cell; CCL, C‐C motif chemokine ligand; CCR, C‐C motif chemokine receptor; CX3CL, C‐X3‐C motif chemokine ligand; CX3CR, C‐X3‐C motif chemokine receptor; CXCL, C‐X‐C motif chemokine ligand; CXCL, C‐X‐C motif chemokine ligand; DAMP, damage‐associated molecular pattern; ECM, extracellular matrix; ICAM, intercellular adhesion molecule‐1; IL, interleukin; JNK, c‐Jun N‐terminal kinase; LFA, lymphocyte function‐associated antigen 1; LPS, lipopolysaccharide; LTA, lipoteichoic acid; Ly6C, lymphocyte antigen 6C; MCAO, middle cerebral artery occlusion; MCP‐1, monocyte chemotactic protein‐1; MIP‐1α, macrophage inflammatory protein‐1 alpha; MMP, matrix metalloproteinase; mRNA, messenger RNA; NF‐κB, nuclear factor‐kappa B; NIHSS, National Institutes of Health Stroke Score; NR4A1, nuclear receptor subfamily 4 group A member 1; P2X4R, purinergic receptor P2X4; PDGF, platelet‐derived growth factor; PSGL‐1, P‐selectin glycoprotein ligand‐1; SDF, stromal cell‐derived factor; TJ, tight junction; TLR, Toll‐like receptor; TNF‐α, tumor necrosis factor‐alpha; VCAM, vascular cell adhesion molecule; VEGF, vascular endothelial growth factor; VLA, very late activation antigen.

### 3.1 CCL2/CCR2

CCL2 is a member of the C‐C chemokine family that plays important roles in cell migration, immune responses, and cell polarization.^51^ It is produced by many cells in the brain, including astrocytes, microglia, endothelial cells, pericytes, perivascular macrophages, and neurons.^52^, ^53^, ^54^, ^55^, ^56^, ^57^ Astrocyte‐derived CCL2 promotes leukocyte infiltration across the BBB, thereby enhancing inflammation.^58^ CCR2 is the main receptor that binds to CCL2 in ischemic stroke. In cancer, CCL2 binds to the T cell receptor, CCR4, to induce T‐cell migration to the target cells or tissues.^59^, ^60^ CCL2 production is mediated by many cytokines, including IFN‐γ, IL‐1/4/6, and TNF‐α.^47^, ^61^ CCL2 is produced by astrocytes and microglia, under anoxic conditions, which are induced by HIF‐1.^6^, ^7^ CCL2 has three domains, the most important being the *N*‐terminal domain, which determines the affinity of CCR2 binding, as demonstrated in an experiment using MCP‐1/3 chimeras.^62^ Changes in this region reduce the chemotaxis of monocytes toward CCL2.

CCR2 is highly expressed on the cell surface of classical monocytes and is involved in monocyte migration. CCR2 has two isoforms: CCR2A and CCR2B. Using both A and B antibodies, CCR2B was identified as the main receptor to which CCL2 binds to initiate signal transduction. CCR2A contributes only 10% of the signaling activity.^63^ CCR2 binds to five proinflammatory chemokines (CCL2, CCL7, CCL8, CCL12, and CCL13), of which CCL2 is the most important in monocytes.^64^ The CCL2/CCR2 axis is involved in monocyte migration, including the migration of monocytes from the bone marrow to the infarcted tissue via the bloodstream. In monocytes, CCL2 only binds to CCR2, and CCR2 is only expressed on the surface of classical monocytes. Evidence from experimental and clinical studies suggests that classical monocytes are the main subset recruited to the brain during an ischemic stroke.^12^, ^13^

The CCL2/CCR2 axis mediates monocyte migration in ischemic stroke and affects the integrity of the BBB. It is also involved in the recovery from ischemic stroke. The mechanism of the CCL2/CCR2 axis in ischemic stroke can be explained as follows. HIF‐1 is produced by astrocytes and other cells, under hypoxic conditions, and enhances CCL2 expression.^58^ CCL2 recruits monocytes and their precursors from the bone marrow to the bloodstream by binding to CCR2. CCL2 is released at sites of inflammation and stored in the glycocalyx, forming a chemokine gradient. CCL2 then recruits circulating monocytes to the inflamed tissue.^15^ CCL2 also regulates the expression of TJ‐associated proteins in brain microvascular endothelial cells (BMECs) by CCR2‐expressing endothelial cells. Plasmin cleaves the *C*‐terminus, which destroys the BBB and allows monocytes to exudate into the parenchyma of the ischemic hemisphere.^48^, ^65^, ^66^ CCL2/CCR2 binding changes the conformation of VLA‐4, which results in high affinity for its receptor VCAM‐1, promoting monocyte adhesion and migration.^18^ CCR2^+^ monocytes promote the differentiation of pericytes into proangiogenic fibroblasts in the brain. As CCR2^+^ monocytes mediate platelet‐derived growth factor (PDGF) beta and PDGF receptor beta messenger RNA (mRNA) expression in ischemic stroke, it can be inferred that monocytes support pericyte differentiation.^67^ We infer that this also enhances tissue repair after ischemic stroke. In CCR2‐deficient mice, monocytes show a delayed increase in proinflammatory mediators, which impairs tissue repair and attenuates the acute inflammatory response.^67^ CCR2^+^ monocytes express high levels of VEGF‐A and are important for vascular sprouting.^68^

The CCL2/CCR2 axis mediates the expression of specific proinflammatory cytokines and regulates the inflammatory response. Upon CCL2 binding to CCR2 on monocytes, intracellular calcium levels increase via activation of the phospholipase C/inositol triphosphate/protein kinase C (PKC) pathway.^69^ PKC activates NF‐κB, which upregulates several genes that produce directional cell motion, promoting cell migration to sites of inflammation.^15^ CCL2/CCR2 binding induces MCP‐1‐induced protein‐1 expression, which initiates the transcription of CCL2, IL‐1, and TNF, thereby creating an inflammatory environment.^47^, ^48^ CCL2 initiates a positive feedback loop of monocyte recruitment.^15^ Other intracellular protein kinases that are known to induce inflammatory responses include extracellular signal‐regulated kinase 1/2, Janus kinase 1/2, mitogen‐activated protein kinase, and phosphatidylinositol 3‐kinase (PI3K).^15^, ^70^, ^71^, ^72^

In macrophages, CCL2/CCR2 modulates LPS‐induced cytokine production, enhance the release of IL‐10, and determine the extent of macrophage polarization through granulocyte–macrophage CSF and macrophage CSF.^51^

### 3.2 CCR4

The chemokine receptor, CCR4, is mainly expressed in T cells, but is also expressed in monocytes, and is important for monocyte migration.^73^ A CCR4 antagonist decreases monocyte migration from the blood to the brain parenchyma in an animal model of neuroinflammation. Kim et al. (2022) showed that CCR4 is involved in the migration of MDMs to the brain and developed a theory that MDMs migrate to the target tissue under the guidance of cytokines released by microglia.

CCR4 also affects macrophage and microglial polarization. M1 macrophage and microglial polarization in ischemic stroke are partially dependent on CCR4 via the chemokine‐like factor 1 (CKLF1)‐mediated NF‐κB pathway. CKLF1 has many functions. Its expression increases during the early phase of ischemic stroke and its main receptor in ischemic stroke is CCR4. Chen et al. (2019) demonstrated the effects of CKLF1 on macrophage and microglial polarization by examining the dose‐dependent increase in the expression of the M1 markers, CD16 and CD32, and the decrease in the expression of the M2 marker, CD206. CCR4 and CCR5 are both CKLF1 receptors. Using CCR4/5 antagonists, it was found that only the CCR4 antagonist had the same effect as CKLF1, suggesting that CCR4 is the main receptor in ischemic stroke.

### 3.3 CCR5

CCR5, a 7‐transmembrane G protein‐coupled receptor, is composed of 352 amino acids and regulates immune responses through ligand interactions.^76^ CCR5 regulates the innate immune response to infection/inflammation, which is closely related to the regulation of leukocyte migration.^77^ Ligand binding enhances the recruitment of monocytes, T helper cells, and microglia. CCR5 is mainly expressed on the surface of intermediate monocytes. CCR5 mediates MDM migration, and its inhibition has been shown to suppress the migration of MDMs to the brain parenchyma in an in vivo model.^74^

CCR5 and its ligand, CCL5, are involved in the pathology of ischemic stroke and monocyte recruitment. CCL5/CCR5 expression increases in the ischemic hemisphere.^78^ CCL5 inhibition reduces Ly6C^+^ monocyte infiltration, attenuating classical monocyte‐mediated inflammation in an ischemic stroke model.^41^, ^78^

CCR5 may also be involved in monocyte migration in other diseases. CCR5 expression is regulated by receptor‐interacting protein 3, which enhances NF‐κB activity by regulating proinflammatory mediators.^79^ In chronic obstructive pulmonary disease, monocyte migration is directed toward CCL5.^80^ In sepsis, CCR5^+^ monocytes migrate to inflammation sites via the bloodstream where they phagocytize and kill bacteria. CCR5 is crucial for monocyte migration from the bone marrow to the bloodstream.^81^

### 3.4 CD36

CD36 is a highly glycosylated class B scavenger receptor that is expressed on many cells, including microglia and monocytes/macrophages. It mediates immune cell migration via the CCL2/CCR2 axis and has many ligands (depending on the context), including apoptotic cells, thrombospondins, fibrillary amyloid‐β, and oxidized lipids.^82^, ^83^ CD36 mediates inflammatory responses upon ligand binding and enhances the phagocytic ability of microglia and monocytes/macrophages.^84^

In ischemic stroke, CD36 expression is upregulated compared to that in normal brain tissues. CD36 mediates monocyte recruitment by regulating CCL2/CCR2 expression in immune cells. In a model of ischemic stroke, CD36 KO mice had reduced CCL2/CCR2 expression. Wild‐type (WT) splenocytes incubated with normal serum had increased CCR2 and CD36 expression compared to those incubated with CD36 KO serum, consistent with the observation that CD36 enhances CCR2 expression in immune cells.^32^ It is conceivable that CD36 mediates monocyte recruitment by regulating CCL2/CCR2 expression. CD36 mediates leukocyte recruitment in neonatal mice, leading to increased inflammatory monocytes and neutrophils, followed by a delayed accumulation of patrolling monocytes early after transient MCAO.^85^

CD36 plays a negative role in the early phase of ischemic stroke and has a restorative function during the resolution phase. CD36 exacerbates brain injury in adult mice with transient MCAO. CD36 KO mice show downregulation of inflammatory mediators (CD68, CSF1, tissue inhibitor of MMP‐1, and TLR4) and upregulation of anti‐inflammatory cytokines (IL‐10, DAMP, and high‐mobility group protein box 1 [HMGB1]). Compared to WT mice, CD36 KO mice have reduced expression of TLR4 after 13 h of reperfusion and reduced numbers of toxic cells.^85^ CD36 is redistributed between the subacute phase and the chronic phase. Cell surface CD36 expression increases 7 days after ischemic stroke with a concomitant reduction in intracellular CD36 expression. CD36 has a restorative function during the resolution phase, suggesting that the mechanism of CD36 depends on the environment and timing of the stroke. CD36 is involved in tissue repair and resolution of inflammation during the resolution phase of ischemic stroke and is also involved in the clearance of cell debris and phagocytosis.

### 3.5 CX3CL1/CX3CR1

CX3CL1 is produced by neurons and plays an important role in neuronal/glial signaling. It is the only member of the CX3C family of chemokines that has only one receptor, CX3CR1.^86^ All monocytes express CX3CR1 at different levels. Classical monocytes express low levels of CX3CR1, whereas non‐classical monocytes express high levels of CX3CR1.^87^ CX3CR1 is not only expressed in monocytes but is also expressed in microglia, where it mediates microglial activity and maintains homeostasis. In CX3CR1‐deficient mice, the CX3CL1/CX3CR1 axis is involved in the progression of atherosclerosis, as CX3CR1 deficiency reduces lesion formation in atherosclerosis.^87^ CX3CR1 is a marker of TGF‐β‐producing monocytes. Human CX3CR1^+^ DCs produce higher levels of TGF‐β than other types of DCs.^88^, ^89^ TGF‐β is a crucial mediator that upregulates CX3CR1 expression on microglia, T cells, and mesangial cells.

CX3CR1 is involved in non‐classical monocyte patrolling along the endothelium. Non‐classical monocytes patrol the endothelium to monitor its integrity, through crawling behavior, and promote tissue repair.^21^, ^90^, ^91^ CX3CR1 mediates monocyte adhesion to the endothelium through the activation of integrins and intrinsic adhesion. Non‐classical monocytes depend on CX3CR1 to patrol the endothelium and LFA‐1 for crawling behavior. In a study using antibody blockade of LFA‐1, detached crawling monocytes were observed in CX3CR1^+/−^ mice.^21^ In cardiovascular disease, reconstituted high‐density lipoprotein reduces CX3CL1/CX3CR1 expression on monocytes and vascular smooth muscle cells.^87^ Therefore, we can infer that reconstituted high‐density lipoprotein modulates the progression of cardiovascular disease by regulating monocyte patrolling behavior.

In ischemic stroke, CX3CR1 affects the volume of the infarcted area, BBB integrity, angiogenesis, and neurological recovery. In a model of ischemic stroke, CX3CR1 KO mice have larger damaged areas than WT and CCR2 KO mice 48 h after stroke. In the chronic phase, CX3CR1 KO mice have minimal brain damage/BBB leakage and increased angiogenesis, which contribute to recovery.^92^ Sex‐related differences are observed where female mice have more severe hippocampal lesions and learning deficits than male mice.^92^, ^93^ CX3CR1 depletion reduces monocyte recruitment, microglial proliferation, and the inflammatory capacity of both cell types.^92^, ^94^ CX3CR1‐depleted monocytes alone may not be sufficient to protect the brain from ischemic stroke.^92^, ^95^

### 3.6 CXCL12 (SDF‐1)

CXCL12 (SDF‐1) is a member of the CXC chemokine family that has a single receptor, C‐X‐C motif chemokine receptor (CXCR) 4, which interacts with all SDF‐1 isoforms. SDF‐1 is involved in the recruitment of monocytes to the infarcted tissue in the late stage of ischemic stroke. In a mouse model of MCAO, SDF‐1 expression is upregulated and persists for >30 days. Monocytes are the main recruited cells 7 days after stroke and become the predominant cells, along with macrophages. Activated astrocytes produce SDF‐1, which mediates CXCR4^+^ bone marrow‐derived cell migration to damaged tissue after ischemic injury. MCP‐1 is an early cytokine that mediates monocyte recruitment. SDF‐1 is involved in the late invasion of the penumbra, as MCP‐1 peaks early and decreases 5 days after stroke.^34^ Therefore, we can infer that astrocyte‐derived SDF‐1 interacts with CXCR4^+^ cells, including monocytes, leading to their recruitment to ischemic tissues.

SDF‐1α regulates monocyte adhesion to BMECs. In neuroinflammation, circulating monocytes adhere to activated BMECs, which express high levels of ICAM‐1, via LFA‐1 integrin. SDF‐1α binding to CXCR4 reduces monocyte binding activity via LYN kinase. Monocytes migrate toward the SDF‐1α gradient, enabling them to cross the BBB.^96^

### 3.7 LFA‐1/ICAM‐1

LFA‐1 is a monocyte‐expressed β‐integrin that binds to ICAM‐1 to mediate monocyte adhesion to the endothelium. ICAM‐1 is a glycoprotein adhesion receptor that is mainly expressed in endothelial cells. Its function is similar to that of chemokines in that it recruits leukocytes from the bloodstream to sites of inflammation.^97^ LFA‐1/ICAM‐1 are required for the patrolling behavior of Ly6C^−^ monocytes on the endothelial surface.^18^ Changes in integrin affinity affect leukocyte patrol along the endothelium. Monocytes require tight binding to the endothelium to perform patrolling behavior. LFA‐1 antibody treatment results in the rapid, prolonged release of monocytes from the endothelial wall, indicating that LFA‐1 is required for crawling.^21^

Soluble ICAM‐1 levels are elevated in patients with ischemic stroke and are associated with stroke outcome. Serum ICAM‐1 levels are higher in patients with poor outcomes than in those with good outcomes. Receiver operating characteristic curve analysis shows that the optimal cutoff value for distinguishing between good and poor outcomes is 129.5 pg/mL (sensitivity, 74%; specificity, 76%).^97^

### 3.8 Ly6C

Ly6C is used to classify murine monocytes into two subsets: Ly6C^hi^ and Ly6C^lo^. Ly6C^hi^ monocytes are proinflammatory, with CCR2 as their receptor. Human (classical) and murine monocytes that express high levels of Ly6C share similar functions. CCR2^+^ monocytes also express high levels of Ly6C.^98^

During the acute phase of ischemic stroke, they infiltrate into the ischemic brain where they inhibit inflammatory and oxidative damage by enhancing M2 macrophage polarization.^98^ CCR2 antagonists that block the binding of CCL2 to CCR2 expressed on monocytes reduce the expression of IL‐10 and the number of circulating and infiltrating Ly6C^hi^ monocytes and prevent increased expression of markers of M2 macrophages (ARG‐1 and YM‐1), suggesting that Ly6C^hi^ monocytes affect macrophage polarization, especially toward the M2 phenotype. Ly6C^hi^ monocytes preferentially differentiate into M1 macrophages, secrete proinflammatory cytokines (IL‐6 and TNF‐α), and enhance T‐cell activation.^98^ Patrolling Ly6C^lo^ monocytes may have little effect on the progression of ischemic stroke. Ly6C^lo^ monocyte depletion does not affect infarct size, cell loss, atrophy, or macrophage and microglial activation at the lesion site.^95^

### 3.9 MMP‐2/9

MMPs are proteinases that participate in ECM degradation. These zinc‐binding endopeptidases are secreted as catalytically latent species that are processed to their activated forms by other proteinases.^31^, ^99^ At least 25 MMPs have been identified to date with different functions in various diseases. MMPs can be divided into five subtypes according to their structural similarity and function, including control of chemokine activity (e.g., CCL2), endothelial repair, innate immunity, and activation of inflammatory cytokines. MMPs are involved in the pathophysiology of ischemic stroke, including BBB degradation,^100^ atherosclerotic plaque maturation, degradation, rupture, and hemorrhagic transformation. MMP‐2/9 are gelatinases that play the most important roles in ischemic stroke.^31^

MMPs are important for cell migration and affect monocyte migration and function. MMPs are secreted by monocytes in different ways (e.g., by binding bacteria to TLR2). MMP expression increases upon monocyte differentiation into MDMs.^101^ MMPs are associated with chemokine secretion (e.g., CCL2).^31^ In ischemic stroke, MMP‐9 activates proinflammatory cytokines and chemokines, such as CXCL8, IL‐1β, and TNF‐α. Therefore, we can infer that MMPs mediate monocyte migration. MMP‐9 mediates monocyte activation through interactions with soluble CD14, leading to reduced responsiveness to LPS in the immune response.^101^ In ischemia, adipocyte fatty acid‐binding protein enhances JNK/c‐Jun activation to promote MMP‐9 transactivation in peripheral monocytes (macrophages and microglia), accelerating the breakdown of the BBB.^37^

MMP‐2/9 levels increase at different time points after ischemic stroke. Early elevations in MMP‐2 levels may be associated with better outcomes, whereas late elevations in MMP‐9 levels may be associated with worse outcomes.^102^ Plasma MMP‐2 levels increase after the onset of ischemic stroke, especially lacunar stroke. There is also evidence that serum MMP‐2 levels do not increase significantly after AIS.^31^ Plasma MMP‐2 levels on admission negatively correlate with NIHSS scores, and patients with better outcomes have higher MMP‐2 levels, suggesting that MMP‐2 has a positive effect on stroke outcomes.^103^ MMP‐2/9 is involved in BBB degradation and MMP‐2 is associated with leukoaraiosis. In a MCAO model, MMP‐2 activation inhibitor‐treated mice have less cell leakage, indicating better BBB integrity. In MMP‐2 KO mice, white matter lesions are less severe than those in WT mice.^31^, ^102^

In ischemic stroke, MMP‐9 is associated with BBB degradation and inflammation, with higher levels of MMP‐9 predicting poor outcomes. This may be explained as follows. MMP‐9 activates proinflammatory cytokines and chemokines, such as CXCL8, IL‐1β, and TNF‐α.^31^ Elevated MMP‐9 levels after ischemic stroke and low levels of MMP‐9 on admission are associated with better NIHSS scores.^5^ In ischemic stroke models, MMP‐9 KO mice have less BBB damage and smaller strokes, and MMP inhibitor‐treated rats have reduced infarct size.^104^ MMP‐9 disrupts BBB integrity by degrading TJs and the ECM to induce cerebral edema.^5^ TJ degradation is inhibited by MMP inhibitors in animal models.^104^ MMP‐9 is associated with ischemia‐induced neuronal death. MMP‐9 inhibitors inhibit oxidative DNA damage and neuronal death after oxygen–glucose deprivation.^105^

### 3.10 NR4A1

NR4A1 functions as a molecular switch in many cellular processes, such as inflammation, proliferation, and apoptosis, and is of fundamental importance to Ly6C^−^ monocytes. NR4A1 is essential for the survival of Ly6C^−^ monocytes and the differentiation of Ly6C^+^ into Ly6C^−^ monocytes. Ly6C^−^ monocytes exhibit apoptosis in the bone marrow of NR4A1^−/−^ mice. Additionally, the conversion of NR4A1^−/−^ Ly6C^+^ monocytes to Ly6C^−^ monocytes is aborted in the bone marrow, blood, and tissues.^11^, ^106^

NR4A1 regulates neuroinflammation in ischemic stroke. In mice, NR4A1 regulates neuroinflammation in cerebral ischemia by interacting with NF‐κB/p65. NR4A1 inhibits inflammatory responses and reduces the infarct volume in ischemic stroke. NR4A1 inhibits microglial M1 polarization and monocyte adhesion and reduces CCL2/7 expression, which is involved in monocyte recruitment.^107^ Infiltrating monocytes differentiate into M2 macrophages in the presence of NR4A1, which has been proven in NR4A1^−/−^ mice. NR4A1^−/−^ mice have more inflammatory M1 macrophages than M2 macrophages.^41^, ^108^ Therefore, we can infer that NR4A1 affects monocyte migration and differentiation.

NR4A1 is involved in the differentiation of Ly6C^lo^ monocytes. NR4A1 deficiency impairs the resolution of inflammation in myocardial infarction. NR4A1 inhibits the infiltration of inflammatory monocytes into the myocardium and the expression of inflammatory mediators by MDMs. NR4A1‐deficient monocytes express higher levels of CCR2 with increased cell mobilization and monocyte infiltration into the infarcted tissue.^108^

### 3.11 P2X4R

P2X4R is an ATP receptor that is highly expressed on immune cells and can modulate the response of myeloid cells, such as peripheral monocytes and resident microglia. Excessive release of ATP from neurons or dead cells overstimulates P2X4R and contributes to ischemic injury. P2X4R is a downstream target of CCL2, which translocates P2X4R to the cell surface.

P2X4R is a double‐edged sword as P2X4R blockade is neuroprotective in the early phase of stroke, but not in the late recovery phase. Compared with WT mice, P2X4R KO mice have reduced infarct volume in the acute phase, but not in the chronic phase. In myeloid‐specific P2X4R KO mice, the mRNA levels of proinflammatory cytokines are elevated in both the acute and chronic phases. P2X4R deficiency leads to a reduction in BDNF.^49^ The mechanism underlying this phenomenon is unknown but may be due to several reasons. First, it has been reported that the activation of P2X4R^+^ cells, especially myeloid cells, promotes the release of proinflammatory cytokines, such as IL‐6/1β and TNF‐α, which play a role in the immune response in the early phase and mediate the release of neuropeptides and growth factors in the chronic/recovery phase. Second, the activation of P2X4R enhances the release of BDNF, which is involved in synaptic plasticity and promotes recovery.^49^

P2X4R is involved in monocyte migration to the brain during ischemic stroke. P2X4R inhibition using the antagonist, 5‐BDBD, reduces monocyte migration to the brain.^9^ CCL2 modulates P2X4R expression to mediate its effect on cells. CCL2 treatment increases the cell surface levels of P2X4R in a concentration‐dependent manner. The CCL2‐induced increase in P2X4R expression is abolished after CCL2 antibody administration, indicating that CCL2 promotes the cell surface expression of P2X4R.^16^ The PI3K/AKT pathway may be involved in this process. p‐AKT expression is significantly increased in response to CCL2 treatment. This increase is reversed by the administration of a CCL2 antagonist, and a PI3K inhibitor decreases the expression of P2X4R.^109^ P2X4R may be involved in the polarization of M1 monocytes, which secrete proinflammatory cytokines and chemokines that degrade TJs between endothelial cells and the BBB.^19^ The mRNA levels of proinflammatory cytokines (IL‐1β/6 and TNF‐α) are elevated in P2X4R KO mice compared to WT mice. The mRNA levels of intracellular cytokines are also elevated in monocytes in myeloid‐specific P2X4R KO mice. These findings suggest that P2X4R may affect the maturation/release of proinflammatory cytokines and may be involved in proinflammatory monocyte polarization.^49^

### 3.12 P‐selectin (CD62P) and CD40L

P‐selectin (CD62P) and CD40L affect the tethering of monocytes and platelets. P‐selectin is a transmembrane adhesion molecule that is expressed on the alpha‐granule membrane of unstimulated platelets and plays an important role in monocyte and platelet adhesion.^110^ CD40L is a transmembrane protein that is expressed on activated platelets. During the acute and subacute phases of ischemic stroke, soluble P‐selectin and CD40L levels increase, and after 90 days, they return to normal. Soluble P‐selectin and CD40L levels in the blood are associated with stroke severity and outcome.^111^

P‐selectin is required for monocyte and platelet tethering and is involved in monocyte recruitment. Upon platelet activation, P‐selectin is phosphorylated and translocated to the membrane where it interacts with PSGL‐1. PSGL‐1 is highly expressed in classical monocytes and is involved in the tethering of monocytes and platelets.^112^ P‐selectin is required for the secretion of CCL2 and IL‐8 by monocytes that are stimulated by activated platelets.^33^ P‐selectin is also involved in monocyte recruitment, which is enhanced by CCL2 secretion after monocyte and platelet adhesion. Tight adhesion occurs after activation and binding to macrophage 1. Cytokine and chemokine secretion (e.g., CCL2 and TNF‐α) are enhanced by the tight adhesion of monocytes and platelets.^87^, ^113^ Therefore, we can infer that adhesion is mediated by P‐selectin and its receptor is involved in monocyte recruitment. Tight adhesion upregulates and activates beta 1/2 integrin and the deposition of platelet‐derived chemokines (CCL5 and CXCL4).^87^ Together, this promotes monocyte recruitment and the formation of a proinflammatory environment.

P‐selectin expression on platelets is increased in patients with ischemic stroke compared to that in healthy controls and is associated with outcome. P‐selectin expression is elevated 24 h after ischemic stroke and is sustained for 7 days. P‐selectin expression and leukocyte counts are higher in patients with poor outcomes compared to those with good outcomes, suggesting that P‐selectin is a biomarker of platelet activation and can predict stroke severity and outcome.^114^

CD40L is a ligand for CD40, which is expressed on monocytes and is structurally related to TNF‐α.^115^ CD40L is upregulated in the ischemic brain and can lead to the formation of platelet–leukocyte aggregates by activating the expression of adhesion molecules (P/E‐selectin and ICAM‐1).^116^, ^117^ After leukocyte involvement in thrombosis, including monocytes, IL‐1/6/8, MIP‐1α, and TNF‐α secretion is triggered by platelet‐derived CD40L interaction with monocyte CD40.^115^ Therefore, we can infer that CD40L promotes the formation of a proinflammatory environment.

CD40L expression on the platelet surface increases in ischemic stroke, and differences in expression may exist between small‐artery and cardioembolic stroke. Patients with ischemic stroke have increased platelet and monocyte activation and upregulated platelet surface expression of CD40L. Patients with small‐artery stroke have higher CD40L expression than those with cardioembolic stroke.^117^

### 3.13 TLR2/4

TLRs are innate immune receptors on the cell surface or inside endosomes that are involved in the inflammatory response.^118^ TLRs are expressed on innate immune cells (monocytes/macrophages, DCs, and neutrophils). They are expressed on the membrane surface and mediate inflammatory responses through the recognition of LPS/lipoteichoic acid (LTA). TLRs recognize cell debris and microbial products from necrotic cells in inflamed tissues, followed by LPS in the case of TLR4, to trigger an inflammatory response.^119^ TLR signaling pathway activates the NF‐κB pathway, promoting the production of proinflammatory cytokines. In the ischemic brain, several cytokines mediate the expression of TLRs, including IFNs, ILs, and exogenous microorganisms. The expression of these mediators is dependent on the NF‐κB pathway.^118^, ^120^ TLR2/4 are the main TLRs studied in ischemic stroke.

TLR2 is associated with the production of inflammatory cytokines by monocytes. TLR2 recognizes LTA and peptidoglycan to initiate downstream responses. In monocytes, TLR2 is upregulated by IL‐1/10 and LPS and downregulated by IFN‐γ, IL‐4, and TNF. During infection, TLR2 may be involved in monocyte migration via the RAC/PI3K pathway. TLR2 expressed on the surface of monocytes stimulates the production of proinflammatory cytokines (IL‐1β/6, MCP‐1, and TNF‐α). This was demonstrated in an experiment using agonist gradients.^119^

TLR2 is involved in inflammation caused by ischemic brain injury. Increased TLR2 expression is associated with poor outcomes in patients with ischemic stroke.^120^ TLR2 is also associated with neuronal apoptosis. TLR2 antibody blockade effectively reduces post‐ischemic neuronal death. In a model of ischemic stroke, TLR2‐deficient mice have a smaller infarct volume and reduced inflammatory cell accumulation (monocyte/macrophage and activated microglia) in the ischemic hemisphere compared to WT mice.^121^ TLR2 binds to HMGB1, an essential DAMP secreted by apoptotic neurons in the ischemic hemisphere that is associated with the severity of neurological impairment in ischemic stroke.^12^, ^118^, ^122^ As TLR2 requires CD36 to initiate an inflammatory response, and CD36 expression is suppressed, inflammation is also suppressed.^118^, ^123^

Classical monocytes express the highest levels of TLR4 among all subsets, supporting their proinflammatory role in the immune response.^124^ TLR4 is involved in signal transduction by CD14 and the LPS/LPS binding protein complex.^119^, ^125^ CD14 is a coreceptor for TLR4, which is activated by DAMPs/pathogen‐associated molecular patterns and is highly expressed in classical monocytes. TLR4 may have less impact on the recruitment of Ly6C^lo^ monocytes. In vitro experiments using TLR agonists showed that late accumulation of Ly6C^lo^ monocytes was promoted by TLR4 compared with other TLRs.^22^, ^23^

TLR4 expression is associated with infarct volume, stroke severity, and functional outcome. In patients with AIS, elevated TLR4 levels in circulating monocytes correlate with increased stroke severity and cytokine levels.^126^, ^127^ TLR4 levels are also associated with ICAM‐1 levels.^14^ TLR4 is involved in the development of infarct volume by binding to its endogenous ligand, HMGB1. HMGB1‐treated TLR4^+^ mice show increased neurological impairment, while TLR4^−^ mice do not differ from WT mice.^118^, ^122^ Myeloid differentiation factor 88 (MyD88) also plays a role in TLR4‐mediated brain injury during ischemia.^127^ MyD88 activates the NF‐κB and activator protein 1 pathways, leading to an inflammatory response and the release of proinflammatory cytokines. MyD88 promotes the interaction of TLRs with TNF‐associated factor 6, which activates TGF‐β‐activated kinase 1 (TAK1) and TAK1‐binding protein ½/3. TAK1 activates mitogen‐activated protein kinase and the IκB kinase complex, which phosphorylates IκB, leading to NF‐κB translocation to the nucleus, where it triggers the expression of proinflammatory genes.^118^, ^128^ In a model of transient cerebral ischemia, TLR4 expression is upregulated, and TLR4‐deficient mice show suppression of proinflammatory cytokines, including cyclooxygenase‐2 (COX‐2), IFN‐β, IFN regulatory factor 1, inducible nitric oxide synthase, and MMP‐9.^14^, ^129^ COX‐2 and MMP‐9 are associated with monocyte infiltration and breakdown of the BBB. TLR4 affects BBB degradation by regulating COX‐2 and MMP‐9, which are implicated in the pathology of ischemic stroke.

### 3.14 VCAM‐1/VLA‐4

VCAM‐1 is an adhesion molecule that binds to VLA‐4 to mediate monocyte recruitment. VCAM‐1 is expressed on endothelial cells and VLA‐4 is expressed on monocytes. VCAM‐1/VLA‐1 mediate monocyte rolling, adhesion, and transmigration. VCAM‐1/VLA‐4 antibody blockade inhibits monocyte recruitment to the brain vasculature during infection.^28^ VCAM‐1 levels increase from the onset to the chronic phase of stroke,^130^ and soluble VCAM‐1 levels are associated with short‐term mortality as non‐survivors have higher levels than survivors.^131^

## 4 CONCLUSIONS

**FIGURE 4:**
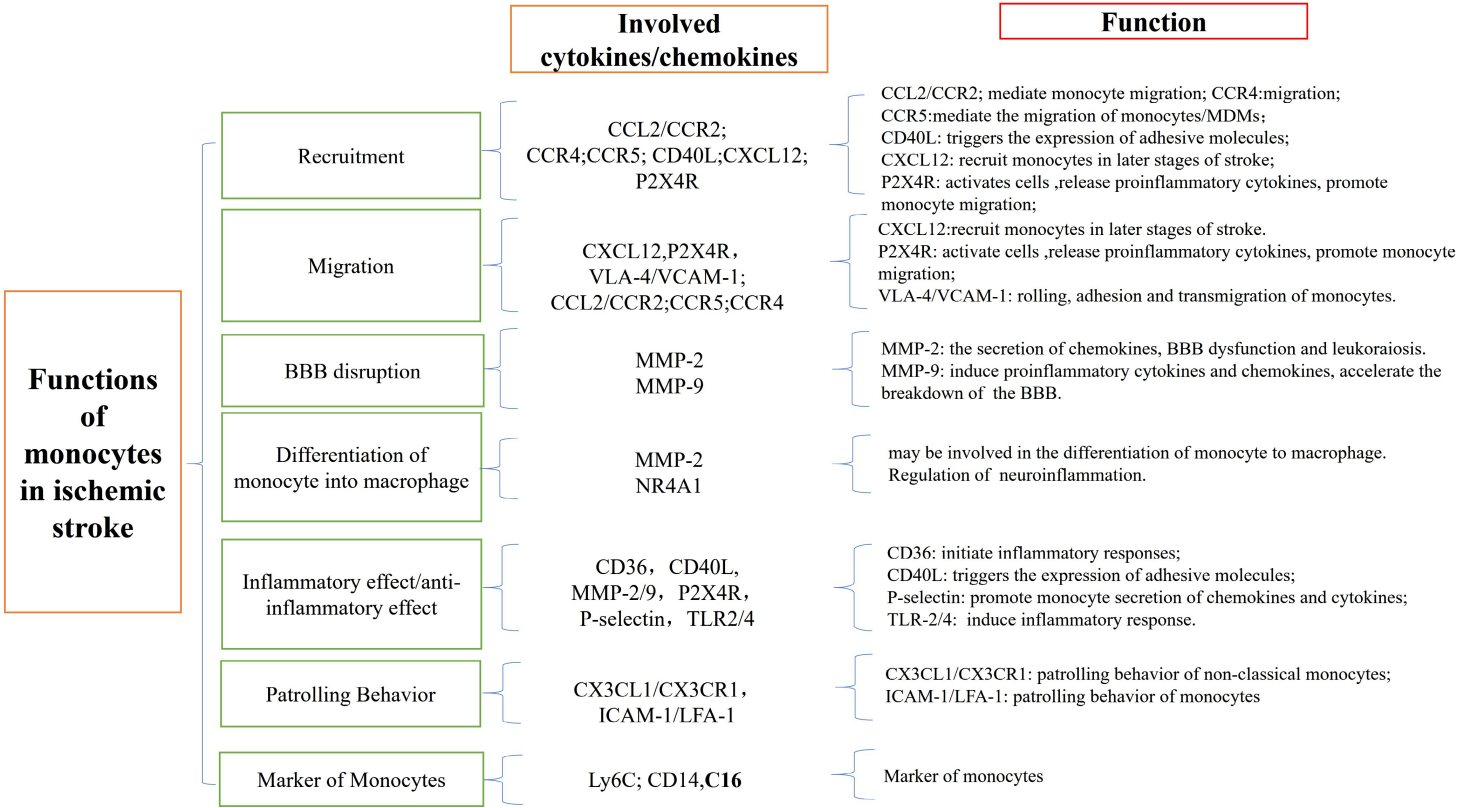
Involved cytokines/chemokines in functions of monocyte in ischemic stroke. Flow chart.

In this review, we focused on the role and mechanism of cytokines and chemokines secreted by monocytes in ischemic stroke. Firstly, we introduced the classification of monocytes. Monocytes can be divided into classical monocytes, intermediate monocytes, and non‐classical monocytes. Classical monocytes are the main producer of inflammatory cytokines in ischemic stroke, which take part in the disruption of the BBB. Intermediate monocytes are in transition from classical to non‐classical monocytes and they are anti‐inflammatory monocytes. Non‐classical monocytes provide immune surveillance by patrolling the endothelium, and they may be protective of the BBB. Secondly, we depicted the role of monocytes in ischemic stroke, including the role of monocytes in the BBB disruption, the differentiation of monocyte to macrophage during ischemic stroke, the recruitment of monocytes in ischemic stroke, as well as the proinflammatory role and the anti‐inflammatory role of monocytes in ischemic stroke. Finally, and most importantly, we described the cytokines and chemokines related to monocytes in ischemic stroke. These include CCL2/CCR2, CCR4, CCR5, CD36, CX3CL1/CX3CR1, CXCL12 (SDF‐1), LFA‐1/ICAM‐1, Ly6C, MMP‐2/9, NR4A1, P2X4R, P‐selectin, CD40L, TLR2/4, and VCAM‐1/VCCL2/CCR2. They are involved in monocyte migration from the bone marrow to the blood and mediate monocyte migration to the inflamed tissue. CCR4 may play a role in monocyte migration, CCR5 mediates the migration of monocytes and MDMs. CD36 interacts with ligands to initiate inflammatory responses and enhances the phagocytosis ability of microglia and monocytes/macrophages. CD40L triggers the expression of adhesive molecules, such as E/P‐selectin and ICAM‐1, leading to the formation of platelet–leukocyte aggregates. CX3CL1/CX3CR1 are required for the patrolling behavior of non‐classical monocytes, activate integrins, and have intrinsic adhesion properties. CXCL12 regulates monocyte adhesion to BMECs. ICAM‐1/LFA‐1 are required for the patrolling behavior of Ly6C^−^monocytes in the endothelium. Ly6C is a classical monocyte marker. MMP‐2 is involved in the secretion of chemokines, such as CCL2 and its level elevates during monocyte‐to‐macrophage differentiation. MMP‐9 is involved in monocyte activation by interacting with soluble CD14. CD36 mediates the monocyte migration through regulation of CCL2/CCR2 and has a restorative function during the resolution phase of inflammation. Nr4a1 is essential for Ly6C^−^ monocyte survival and the transition from Ly6C^+^ to Ly6C^−^ monocytes and may impact the differentiation of monocytes into macrophages. P2X4R promotes monocyte migration and may be involved in the release of proinflammatory cytokines. P‐selectin participates in monocyte–platelet tethering, and promotes monocyte secretion of CCL2, IL‐8, and TNF‐α. P‐selectin and CD40L affect the tethering of monocytes and platelets. P‐selectin is involved in monocyte recruitment. TLR2 and TLR4 are associated with the production of inflammatory cytokines by monocytes, TLR4 expression is associated with infarct volume, stroke severity, and functional outcome. VCAM‐1 binds to VLA‐4 to mediate monocyte recruitment and VCAM‐1 levels increase in ischemic stroke. The flow chart of cytokines/chemokines involved in the function of monocytes in ischemic stroke is in Figure 4.

In conclusion, this review introduces the monocyte‐related cytokines in cerebral ischemic stroke to lay the foundation for the study of the mechanisms of monocyte function in ischemic stroke and provides new targets for the treatment of ischemic stroke.

## AUTHOR CONTRIBUTIONS

Jue Wang designed the concept and the idea of this article. Meiling Bai designed and wrote this manuscript and designed the picture and table. Ruize Sun, Bin Cao, Juan Feng, and Jue Wang gave constructive advice and participated in proofreading this article. All authors contributed to the article and approved the submitted version. All authors have read and approved the manuscript.

## CONFLICT OF INTEREST STATEMENT

The authors declare no conflicts of interest.

## FUNDING INFORMATION

This work was supported by the National Natural Science Foundation of China [grant number 82271353 to JW]. The sponsor had no role in the study design; in the collection, analysis, and interpretation of data; in the writing of the report; in the decision to submit the article for publication.

## Data Availability

Data sharing is not applicable to this article as no new data were created or analyzed in this study.
